# Proteomic signatures of myeloid derived suppressor cells from liver and lung metastases reveal functional divergence and potential therapeutic targets

**DOI:** 10.1038/s41420-021-00621-x

**Published:** 2021-09-04

**Authors:** Nicholas A. DaSilva, Benjamin J. Barlock, Prajna Guha, Chandra C. Ghosh, Catherine E. Trebino, Jodi L. Camberg, Steven C. Katz, David C. Rowley

**Affiliations:** 1 University of Rhode Island, Department of Biomedical and Pharmaceutical Sciences, Kingston, RI USA; 2 Roger Williams Medical Center, Immuno-oncology Institute and Division of Immunotherapy, Department of Medicine, Providence, RI USA; 3 Boston University School of Medicine, Department of Surgery, Boston, MA USA; 4 University of Rhode Island, Department of Cell & Molecular Biology, Kingston, RI USA

**Keywords:** Proteomics, Cancer microenvironment, Immunosuppression, Target identification

## Abstract

Myeloid-derived suppressor cells (MDSCs) promote immunosuppressive activities in the tumor microenvironment (TME), resulting in increased tumor burden and diminishing the anti-tumor response of immunotherapies. While primary and metastatic tumors are typically the focal points of therapeutic development, the immune cells of the TME are differentially programmed by the tissue of the metastatic site. In particular, MDSCs are programmed uniquely within different organs in the context of tumor progression. Given that MDSC plasticity is shaped by the surrounding environment, the proteomes of MDSCs from different metastatic sites are hypothesized to be unique. A bottom-up proteomics approach using sequential window acquisition of all theoretical mass spectra (SWATH-MS) was used to quantify the proteome of CD11b^+^ cells derived from murine liver metastases (LM) and lung metastases (LuM). A comparative proteomics workflow was employed to compare MDSC proteins from LuM (LuM-MDSC) and LM (LM-MDSC) while also elucidating common signaling pathways, protein function, and possible drug-protein interactions. SWATH-MS identified 2516 proteins from 200 µg of sample. Of the 2516 proteins, 2367 have matching transcriptomic data. Upregulated proteins from lung and liver-derived murine CD11b^+^ cells with matching mRNA transcriptomic data were categorized based on target knowledge and level of drug development. Comparative proteomic analysis demonstrates that liver and lung tumor-derived MDSCs have distinct proteomes that may be subject to pharmacologic manipulation.

## Introduction

The therapeutic approach for treating primary and metastatic solid tumors has generally been agonistic to the site of disease with respect to chemotherapeutic regimens [1, 2]. Tumors are complex biologic systems which are influenced by surrounding normal organ parenchymal and stromal cells. The tumor microenvironment contains a diverse array of cell types including immature myeloid cells (MDSCs), T cells (Tregs, CD8^+^, CD4^+^), natural killer cells, dendritic cells, macrophages, neutrophils, and innate lymphoid cells. MDSCs are a heterogenous population of immature myeloid cells which are Gr-1^+^ and CD11b^+^ [3, 4]. Like other immune cell populations, MDSCs are programmed uniquely within different organs [5]. In particular, liver metastases orchestrate a phenotypic and functional program among MDSC that results in strong suppression of immune responses. Within the last decade, MDSCs have proven to be a major factor in diminishing anti-tumor immunity and the efficacy of immunotherapies for patients being treated for hematologic and solid tumors alike [4]. Hence, depletion, functional modulation, or terminal differentiation of MDSCs are attractive strategies for improving immunotherapy efficacy in tumors and lymphoid tissues [6, 7]. Strategies for targeting MDSCs should take into account the unique biological environment of the site of disease in patients with metastatic solid tumors.

MDSCs can be categorized based on morphology and as either granulocytic (G) (i.e., polymorphonuclear (PMN), CD11b^+^Ly6G^hi^Ly6C^lo^) or mononuclear (M) (i.e., monocytic, CD11b^+^Ly6G^lo^Ly6C^hi^) MDSCs [3, 8]. While the ratio of G-MDSCs to M-MDSCs in lymphoid, spleen, and peripheral blood of patients is unique across multiple cancer types (e.g., breast, lung, and colon), G-MDSCs are far less immunosuppressive as compared to M-MDSCs in either lymphoid or tumor sites and are observed in all cancers [9–11].

MDSCs exert their immunosuppressive abilities during the establishment of a pre-metastatic niche in distant tissues. The responsible mechanisms include suppression of T-cell function, influencing regulatory T-cell (Treg) development/recruitment, and modulating NK cell activity [12–15]. MDSCs also aid in tumor establishment and sustainment via the production of free radical generating species (O_2_, NO, PNT, H_2_O_2_), nitration of chemokines/cytokines, blockade of CD8^+^ T cells with tumor cells, and depletion of amino acids essential to T-cell proliferation (i.e., arginine, cysteine, and tryptophan) [12–14, 16, 17]. While MDSCs were thought to be phenotypically and functionally identical in both primary and metastatic tumor sites, clinical evidence suggests this may not be the case [2, 3].

Tumor localized MDSCs rely heavily upon immunosuppression and tissue remodeling through matrix metalloproteases (e.g., Mmp9) to maintain the tumor microenvironment, support angiogenesis, and promote additional tumor metastases [10, 18–20]. In murine metastatic lung tumor models, MDSCs were found to populate the tissue as early as 2 weeks prior to tumor formation; secrete IL-6, S100A8/A9, VEGF, and IL-10 in order to establish the tumor; and recruit additional MDSCs to the lung [21]. When co-culturing lung tumor-derived M-MDSCs or G-MDSCs from mice with 4T1 breast carcinoma cells, tumor cell proliferation increased with augmented expression of S100A8/A9, Mmp8, Lyz2, Fpr, Ccl3, and Tgfb2. This unique expression signature also corresponded with a poor survival prognosis in humans with various cancers [22]. Recently, other murine metastatic models confirmed increased numbers of G-MDSCs in LM and elevated levels of STAT5 expression in LuM-MDSCs. STAT5 rather than STAT3 was found to play a significant role in LuM-MDSC proliferation [5].

Despite displaying lower levels of the major ROS generating enzymes arginase-1 (Arg1) and inducible nitric oxide synthase (iNOS) commonly found in immunosuppressive MDSC populations, pro-angiogenic (i.e., tumorigenic) VEGF transcript levels were elevated in LuM-MDSCs than in LM-MDSCs [5]. In LM-MDSCs, the STAT3 associated cytokine, GM-CSF, is a critical driver in MDSC expansion and proliferation. Moreover, the STAT3-JAK2 axis and its inhibition induce Fas-mediated apoptosis in LM-MDSCs [23]. PD-L1 overexpression is also heavily relied upon in order to abolish anti-tumor responses [24]. In particular, the liver is often the most frequently observed organ to harbor neoplastic lesions thanks to its unique architectural and functional features. Liver-specific microcirculation in sinusoidal cells as well as immune regulation via MDSCs, lymphocytes, neutrophils, Kupffer cells, activated hepatic stellate cells, and hepatocytes can all support the pre-metastatic niche for circulating tumor cells [25].

Current knowledge suggests that while MDSCs overexpress key immunosuppressive mediators rather than generating novel mediators, tumor localized MDSCs also play a critical role in the prevention of tumor rejection and thus promote tumor growth through other mechanisms [10, 20]. Therefore, identifying and characterizing unique proteomic signatures from populations of MDSCs in metastatic tumor sites may inform the design of new strategies to modulate these cells for improved immunotherapy outcomes in an organ-dependent manner. In this study, we compared the proteomes of MDSCs derived from orthotopically induced liver and lung tumors in mice, hypothesizing that MDSC proteomes would differ based on tumor location. A comprehensive, label-free methodology, termed sequential window acquisition of all theoretical mass spectra (SWATH-MS) analysis, was used to identify and quantify MDSC proteins from lung and liver tumors. This proteomic analysis was then matched with transcriptomic data to reveal the organ-specific differences in MDSC gene transcription and protein production.

## Results

### Protein quality check, principal component analysis, and hierarchical clustering demonstrate distinct LM and LuM proteomes

**Fig. 1:**
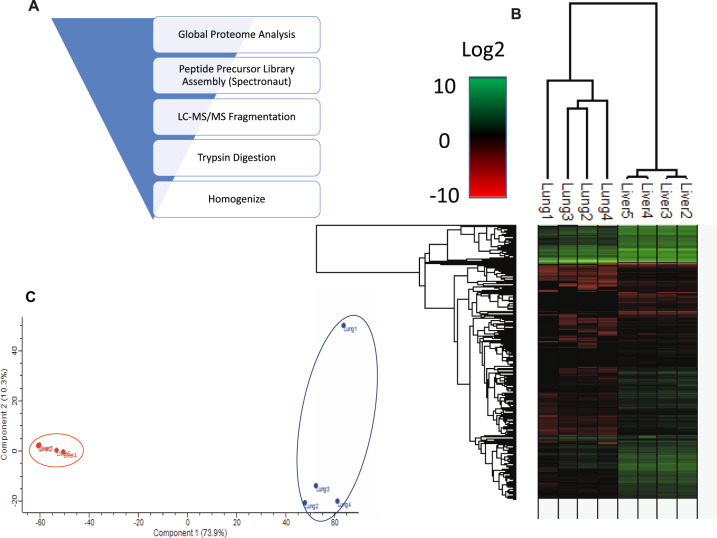
LC-MS/MS proteomics workflow, principal component analysis, and hierarchical clustering. SWATH-MS bottom-up proteomics was used to quantify protein abundance using the total protein approach from data handled by Spectronaut (**A**). Euclidean distance hierarchical clustering (**B**) showed tissue metastasis-specific proteomes with liver metastases appearing to contain more proteins than those from lung. Principal component analysis further confirmed tissue-specific clustering based on their quantified proteome (**C**).

Raw spectral counts were handled using Spectronaut in order to generate a comprehensive and reproducible proteomic library (Fig. 1A). Following Spectronaut’s internal normalization algorithm [26], 2516 proteins were identified across all samples (*n* = 4 per tissue type) with 99.8% recovery (*Q* value ≤ 0.01). Principal component analysis of each sample type and their respective protein abundance values fell into two distinct sample-specific clusters (Fig. 1C). As expected, hierarchical clustering of log2 transformed protein abundance data confirmed a distinct LM and LuM proteomic signature (Fig. 1B). As a whole, LM samples appeared to be less variable and showed greater protein enrichment than LuM samples.

### Identification of upregulated liver and lung-specific proteins, core MDSC proteins, and comparisons between transcriptomic data

**Fig. 2:**
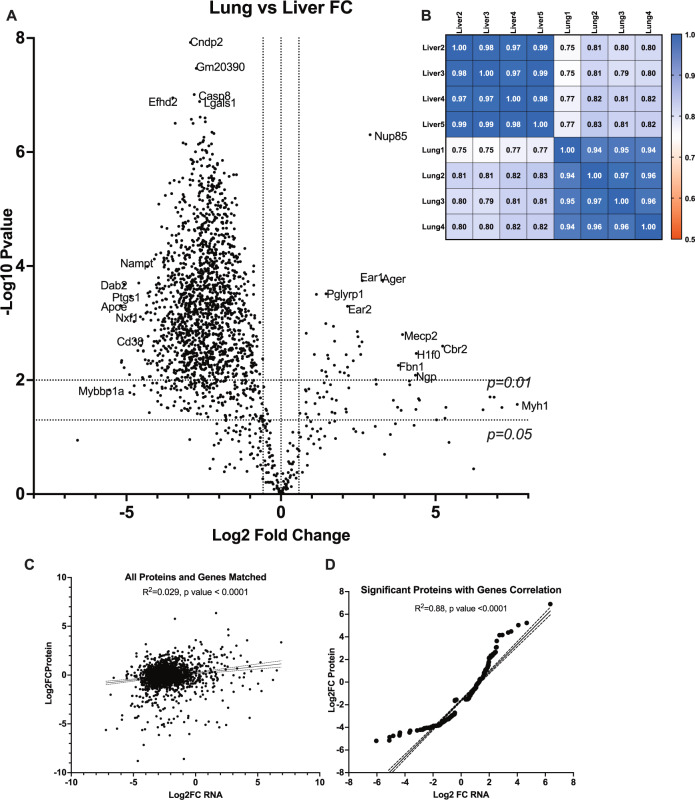
Comparisons of the global proteomes of LuM and LM and their matching transcriptomic data reveal tissue-specific proteomic signatures. Volcano plot (**A**) and Spearman correlation matrix (**B**) of all 2516 Proteins comparing proteomic changes between LuM and LM. Matching RNA transcriptomic data was found for 2367 proteins whereas significantly up or downregulated proteins have 317 matching transcriptomic data. Correlation of all 2367 matching proteins and mRNA was low (**C**) but differentially expressed significant proteins (**D**) correlated well with transcriptomic data.

Over 16,000 genes were previously sequenced from LM and LuM samples [5]. By comparison, 2516 proteins were identified with 2367 proteins having matching mRNA data [5]. A total of 809 differentially expressed proteins were identified after transformation of the protein abundances from pmol per mg of protein to a Log2 scale and then calculating the fold change of lung over liver proteins and setting statistical thresholds, where proteins with *p* values < 0.05 were considered for future analyses (Fig. 2A). Proteins with an FC near 0 (within Log2FC −0.58 and 0.58) were identified as core proteins, i.e., those consistently expressed in both LM and LuM samples (Fig. 2A). Spearman correlations further showed LM and LuM samples were correlating with their respective tissues yet still maintained some concordance between LM and LuM samples (Fig. 2B). Of the 809 significant proteins identified by Spectronaut, 317 matched with mRNA data from RNASeq of these same tissue sample types. Moreover, the correlation between matching transcriptomic and proteomic log2FC values was low (*R*^2^ = 0.029, Pearson *r* = 0.1706) as typically observed in other comparative studies (Fig. 2C) [27–29]. However, statistically significant proteins with matching mRNA data showed good correlation (Fig. 2D). Using western blotting, Apoe was validated to be liver-specific as compared to Lung CD11b^+^ cell lysate (Fig. S1).

### Identification of biological function and pharmacologic intervention of LuM and LM-specific proteins

**Fig. 3:**
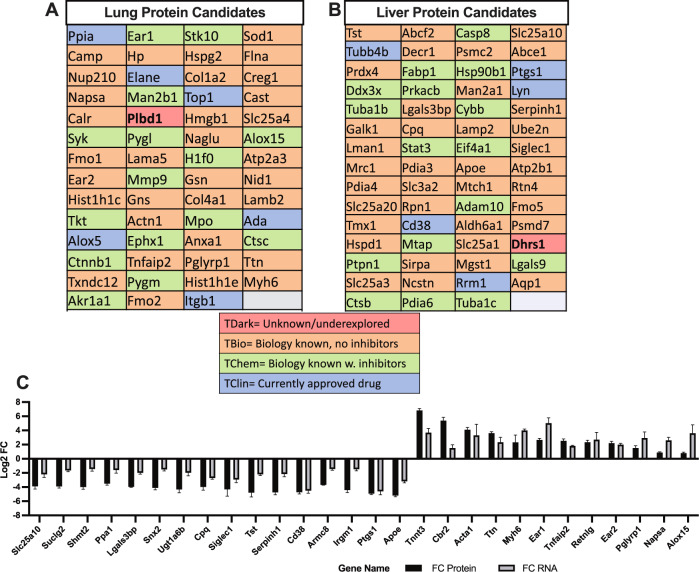
Potentially druggable protein targets from LM and LuM CD11b^+^ cells with correlating transcriptomic data. The drug–gene interaction database (DGIdb) and PHAROs drug–target development data mining platforms highlight possible targets for future validation experiments and translational studies based on current protein knowledge (**A** and **B**). Positive correlating proteomic and transcriptomic data (**C**) show possible targets for future MDSC modulating strategies for LuM- or LM-specific MDSCs. Data shown *n* = 4 per tissue type mean +/− SEM.

PHAROS database mining of LuM sample proteins (Fig. 3A) showed six proteins had clinically approved inhibitors (TClin). These potential targets included peptidyl-prolyl cis-trans isomerase A (Ppia), neutrophil elastase (Elane), topoisomerase 1 (Top1), adenosine deaminase (Ada), arachidonate 5-lipoxygenase (Alox5), and integrin beta 1 (Itgb1), and hence could serve as a fast-track approach to designing future validation and translational studies. Over thirty proteins were also classified as having a known biological role with no known pharmacologic inhibitors (TBio), such as superoxide dismutase 1 (Sod1), or as having small molecule or biologic inhibitors (TChem), such as matrix metalloproteinase 9 (Mmp9) (Fig. 3A). Notably, only one protein, phospholipase b-like 1 (Plbd1), was classified as having an unknown function or any chemical/biological inhibitors (Tdark) and could represent a new avenue to pursue in MDSC biology.

In LM (Fig. 3B), tubulin beta 4B chain (Tubb4b), cyclooxygenase 1 (Ptgs1), tyrosine-protein kinase lyn (Lyn), ADP-ribosyl cyclase/cyclic ADP-ribose hydrolase 1 (CD38), and ribonucleoside-diphosphate reductase large subunit (Rrm1) were also identified as having clinically approved treatments. Dehydrogenase/reductase SDR family member 1 (Dhrs1) was another enriched protein specific to LM that was identified as having relatively little-known regarding biology or function (TDark). Conversely, 16 LM-specific proteins were classified as having a known biological role with chemical and or biologic inhibitors/modulators.

### Liver and lung-specific proteins linked to ER stress, ECM remodeling, and necroptosis

**Fig. 4:**
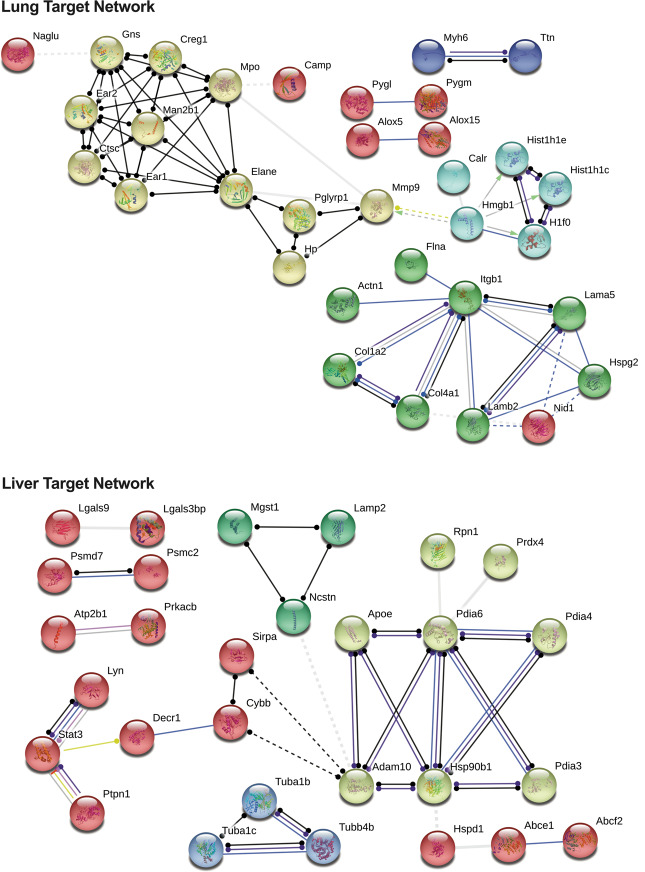
STRING pathway analysis of LM and LuM CD11b^+^ druggable proteomic targets. Druggable targets from the previous workflow were subject to pathway analysis using the data mining platform STRING. Nodes with multiple interactions within key pathways may serve as regulatory focal points of a given signaling pathway.

**Fig. 5:**
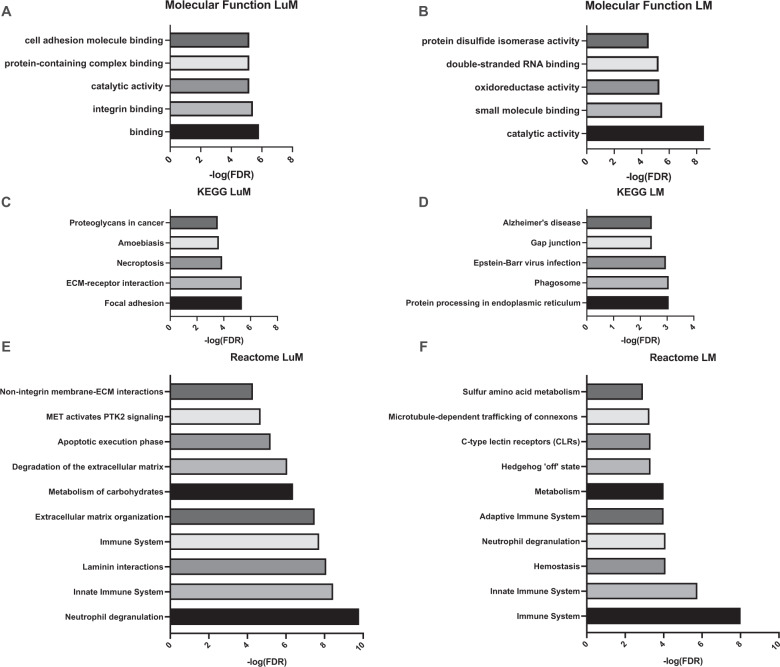
Molecular function and pathway (KEGG and REACTOME) annotations of LM and LuM CD11b^+^ cells. LuM and LM proteins were annotated for Molecular function (**A**, **B**), KEGG (**C**, **D**), and Reactome Pathways (**E**, **F**), respectively. Druggable proteins with matching transcriptomic were annotated for function and major pathway associations using STRING. False discovery rate threshold was set to <1%.

Using the output from mining proteins in the DGIdb, 55 proteins from lung and 59 proteins from LM were further analyzed using STRING (Fig. 4). For LM, Hsp90b1, Pdia6, Stat3, Lyn, Ptpn1 could potentially be modulated. Conversely, Man2b1, Elane, Itgb1 for lung-derived CD11b^+^ cells represent possible proteomic lynchpins regulating cell survival, neutrophil degranulation, MDSC recruitment, metabolism, and inflammation. STRING protein network associations between the 55 LuM proteins showed KEGG pathways involving focal adhesion (*q* = 4.38 × 10^−6^), ECM receptor interaction (*q* = 4.57 × 10^−6^), and necroptosis (*q* = 1.3 × 10^−4^) (Fig. 5C). Furthermore, REACTOME pathway analysis found overlap with neutrophil degranulation (*q* = 1.62 × 10^−10^), innate immune system (*q* = 3.56 × 10^−9^), and laminin interaction (*q* = 8.13 × 10^−9^) pathways (Fig. 5E). Pathway analysis using STRING of the 59 proteins in LM samples demonstrated pathways implicated in response to chemical stress, phagosome formation (*q* = 8.7 × 10^−4^), and endoplasmic reticulum processing stress (*q* = 8.7 × 10^−4^) (Fig. 5B). Further, just as in LuM samples, general immune system (*q* = 9.82 × 10^−9^), innate immune system (*q* = 1.76 × 10^−6^), and neutrophil degranulation (*q* = 7.96 × 10^−5^) pathways were significantly enriched in LM (Fig. 5F).

## Discussion

Recent findings from our labs demonstrate that the TME in liver and lung differentially shape MDSC function [5]. Specifically, pSTAT3 and pSTAT5 signaling are major drivers of MDSC expansion and immunosuppression in liver and lung, respectively. A critical finding was that the liver is capable of a reversible yet specific role in the immunosuppressive programming of myeloid cells. In this study, we sought to compare and comprehensively quantify proteins from CD11b^+^ cells of the TME to further explore differential programming of MDSC populations from liver and lung tissues. Findings from this proteomic approach further support the notion that organ-specific MDSC targets may be exploited as part of a treatment regimen to enhance immunotherapy for solid tumors.

Previous proteomic studies have examined MDSCs from circulation, spleens, exosomes, and different tumor types (metastatic and non-metastatic tumors), employing various MS-based proteomics methodologies [30, 31]. For example, Boutté et al. [30] employed a semi-quantitative proteomics approach on splenic MDSCs from BALB/c mice orthotopically injected with non-metastatic (67NR) and metastatic (4T1) mammary tumors. In total, 2814 proteins were identified using MUDPIT followed by IDpicker for spectral counts of peptides, followed by Webgestalt and Pathway studies for data processing. With these methods of MDSC harvesting, isolation, sample preparation, and LC-MS/MS analysis in mind, these previous studies sought to examine proteomic changes in MDSCs outside of the tumor site. These previous studies identified cell adhesion, immune system processing, and coagulation as relevant to MDSC biological roles, consistent with this current study.

The SWATH methodology and bottom-up proteomics approach is well established, and by combining high-resolution LC-MS/MS analysis with Spectronaut data processing in tandem with the Uniprot protein database, this workflow has provided new insights into the variability of MDSC proteomes [32–34]. PCA and hierarchical clustering of all proteins between LM and LuM cells support the hypothesis that the TMEs in liver and lung tissues harbor distinct populations of MDSCs with unique proteomes. Further comparisons of the proteomic data with previously acquired RNASeq data showed an overlap of ~2300 proteins with mRNA transcriptomic data. The overall correlation between Log2FC of RNA and Log2FC of protein abundance was low as seen in proteomic/transcriptomic studies from other cell types [28, 29].

Pathway analysis revealed that significantly upregulated LM proteins that were considered to be both druggable and had a matching mRNA dataset were implicated in responding to chemical or inflammatory stress. These included Lyn and Ptpn1 which transcriptionally regulate and post-translationally modify STAT3 [35–37]. Protein folding and processing in endoplasmic reticulum, a likely survival mechanism of MDSCs to manage misfolded proteins due to hypoxic, nutrient-deprived, or inflammation-rich TME conditions, was another significant LM pathway with potential targets, including Hsp90b1 and Pdia6 [38–41]. Notably, LM-specific targets such as the cytoskeletal proteins Tubb4b, Tuba1c, and Tuba1b were found to overlap with activities of the phagosome/exosome pathways [42]. The formation, regulation, and release of exosomes, which traffic signaling molecules, transcription factors (microRNA), and proteins, could be seen here as a result of recruiting additional MDSCs to the metastatic tumor site thus supporting the metastatic niche [42–44]. Another commonality with these targets is their role in innate immune function and neutrophil degranulation [4, 45, 46].

Using PHAROs to help distinguish novel from clinically exploited proteins, Lyn, Ptgs1, Tubb4b, CD38, and Rrm1 were identified as potential drug targets where clinically approved inhibitors already exist and may have applications in the depletion of MDSCs or modulation of their immunosuppressive activity. Specifically, Ptgs1 (or COX-1), which is overexpressed in LM compared to LuM, has over 30 approved inhibitors (e.g., celecoxib, naproxen, ibuprophen, diclofenac, etc.) and over 200 ligands (e.g., resveratrol). Notably, one study demonstrated that aspirin-treated COX-1 knockout mice had fewer MDSCs in both immune tissues and at the inflamed site (lung). Further, this reduction in immunosuppression may be attributed to the inhibition or deletion of COX-1 and downregulation of Arg-1 [47].

CD38 and Lyn are additional druggable targets that are overexpressed in LM-MDSCs. Inhibition of CD38 may halt the recruitment, differentiation, or immunosuppressive function of MDSCs and even abrogate tumorigenesis [48–50]. Lyn, a SRC family kinase, which is inhibited by dasatinib, phosphorylates, and activates STAT3 and is regarded as a driver of MDSC differentiation and expansion [35, 36, 51]. Moreover, overexpression or gain of function mutations in Lyn may lead to abnormalities in myeloid-derived cells [52]. Inhibition of Lyn in mice reduced ROS and MDSC accumulation but also reduces tumorigenesis in a head and neck cancer model [51].

As with LM-MDSCs, immune system-centric pathways were observed as potentially druggable targets when analyzing the upregulated proteins in LuM samples. Specifically, focal adhesion and ECM receptor pathways involve Itgb1, Col1a2, Col4a1, Lama5, and Lamb2. Necroptosis pathway enrichment was due to the presence of Hmgb1, Alox15, Pygl, and Pygm. Notably, neutrophil degranulation was also implicated in LuM-MDSCs and included proteins such as Mmp9, Mpo, Elane, Alox5, Pygl, Man2b, Ear1, Ear2, Clsc, Creg1, Gns, and Pglyrp1. Itgb1, Elane, Alox5, Ada, Top1, and Ppia were identified as proteins that have known FDA-approved drugs.

In particular, studies show that integrins are essential in endothelial trafficking and establishing the tumor microenvironment [53–55]. Furthermore, Itgb1 is found on MDSCs and bone marrow-derived immune cells which promote tumor inflammation [56]. Neutrophil elastase (Elane), which is responsible for NK, monocyte, granulocyte, and neutrophil function and chemotaxis, can be modulated with the inhibitor sivelestat [57–60]. As described by Lerman et al. [57], treatment of xenograft tumor-induced mice with sivelestat depleted Gr-1^+^ MDSCs and abrogated tumor growth.

By combining mass spectrometry-based proteomics and drug–target databases, we were able to comprehensively quantify proteins from LM- and LuM-MDSCs to prioritize possible protein targets for future inhibitor development. In this study, we show that LM- and LuM-MDSCs have unique proteomes that contribute to their organ-specific programming and propensity to promote tumor growth and inflammation. These unique proteomes may be exploited to develop new inhibitors or to repurpose currently marketed inhibitors to deplete or functionally modify MDSCs found within liver and lung metastatic sites. Ultimately, this heterogeneity suggests that tissue-specific therapies may be possible to improve immunotherapy outcomes and urges additional biological validation of these targets in vivo.

## Materials and methods

### Chemicals and reagents

TPCK-treated trypsin, trypsin-digested β-galactosidase, and mass spectrometer tuning solution were purchased from SCIEX (Framingham, MA). Acquity UPLC Peptide BEH C18 analytical column and VanGuard pre-columns were procured from Waters Corp. (Waltham, MA). 1,4-Dithiothreitol (DTT) was obtained from Roche Diagnostics (Indianapolis, IN). Bovine serum albumin, sodium deoxycholate, and iodoacetamide (IAA) were procured from Sigma Aldrich (St. Louis, MO). MS grade acetonitrile and formic acid were purchased from ThermoFisher Scientific (Waltham, MA).

### Mice, LM LuM in vivo model and MDSC isolation

C57BL/6J, B6.SJL-Ptprca Pepcb/BoyJ (CD45.1) male mice (6–8 weeks old) obtained from Jackson Laboratories were bred and maintained under pathogen-free conditions at the Roger Williams Medical Center (RWMC) animal facility. MDSCs were isolated for RNA sequencing or SWATH-mass spectrometry proteomics as reported previously [5].

### MDSC homogenate preparation

Using previously published preparation methods [32], ~9–16 × 10^6^ CD11b^+^ lung MDSCs (LuM) and CD11b^+^ liver MDSCs (LM) were collected from tumor burdened mice (*n* = 4 per tumor type) and homogenized in 300 µL of homogenization buffer (8 M urea and 50 mM triethylammonium bicarbonate in MilliQ ddH_2_O) using a bead homogenizer (Omni Bead Ruptor, Kennesaw, GA). Supernatant was collected after spinning at 1000 × *g* for 5 min. Total protein concentration was determined using a Pierce BCA protein assay kit (ThermoFisher Scientific, Waltham, MA).

### In-solution trypsin digestion

Protein digestion was conducted as described previously with modifications [32, 61]. In this study, cell homogenate (*n* = 4 per tissue type; ~200 µg protein), along with an internal control of 2 µg bovine serum albumin (BSA), were denatured with 25 µL DTT (100 mM) at 35 °C for 30 min in a shaking water bath (100 rpm). Samples were then alkylated in the dark with 25 µL IAA (200 mM) at room temperature for 30 min. After alkylation, samples were concentrated using the cold water, methanol, and chloroform (1:2:1, v/v/v) precipitation method followed by centrifugation at 12,000 rpm, 5 min at 10 °C. Ice-cold methanol was used to wash the protein pellet. The pellet was then suspended in 200 µL of 50 mM ammonium bicarbonate (pH ~8) containing 3% w/v sodium deoxycholate (DOC). Further, 135 µL of the sample was then spiked with TPCK-treated trypsin (10 µg) and samples were transferred into digestion tubes (PCT MicroTubes, Pressure Biosciences Inc., Easton, MA). The barocycler was run at 35 °C for 75 cycles with a 60 s pressure-cycle (50 s high pressure, 10 s ambient pressure, 25 kpsi). Following the first run, 10 µg trypsin was added to each sample and the barocycler was run again at the above settings. After the barocycler, 15 µL of 5%v/v formic acid (1:1 LCMS grade acetonitrile and H_2_O) was added to 125 µL of digested peptides sample in order to precipitate DOC. Supernatant was collected after samples were centrifuged at 10,000 rpm for 5 min at 10 °C. Subsequently, 25 µL of the digested peptide sample was injected and analyzed using LC-MS/MS.

### LC-MS/MS analysis

Data-independent analysis (DIA) was performed in positive ionization mode using a DuoSpray™ ion source on a Sciex 5600 TripleTOF™ mass spectrometer (Sciex, Framingham, MA, USA). Separation was achieved using an Acquity UPLC H-Class system (Waters Corp., Milford, MA, USA). Ion spray voltage floating (ISVF) was kept at 5500 V while the source temperature (TEM) was 500 °C. Gas 1 (GS1), gas 2 (GS2), and curtain gas (CUR) were set to 55, 60, and 25 psi, respectively. Declustering potential (DP), collision energy (CE), and collision energy spread (CES) were set at 120, 10, and 5, respectively. During the survey scan, all the ions with a charge state of 2 to 4, mass range of *m/z* 300–1250, and exceeding 25 cps were used for MS/MS analysis. Former target ions were excluded for 8 s and the mass tolerance for TOF-MS was 50 mDa with a 100 ms accumulation time. For the product scan, data were acquired from 100 to 1250 *m/z* with an accumulation time of 75 ms and a total cycle time of 3.5 s. Production analysis was done under dynamic accumulation and rolling collision energy dependent on the *m/z* of the ion. All the parameters for SWATH-MS data acquisition were similar as described above except the following: source temperature (TEM) was 400 °C, GS1 was 55 psi, and TOF masses were collected from *m/z* 300 to 1500. The total cycle time for SWATH acquisition was 3.95 s. SWATH data was acquired (*m/z* 400–1100) over 70 SWATH windows per cycle with a window size of *m/z* 10. Chromatographic separation was achieved over 180 min gradient method at 100 µL/min on an Acquity UPLC Peptide BEH C18 column (2.1 × 150 mm, 300 Å, 1.7 µm) preceded by an Acquity VanGuard pre-column (2.1 × 5 mm, 300 Å, 1.7 µm). Mobile phase A was H_2_O (0.1% formic acid) and mobile phase B was acetonitrile (0.1% formic acid).

Gradient conditions were 98% A from 0 to 5 min, 98 to 70% A from 5 to 155 min, 70 to 50% A from 155 to 160 min, 50 to 5% A from 160 to 170 min, and 5% A to 98% from 170 to 180 min. The gradient was held at initial conditions until the end of the run to equilibrate the column before the start of the next run. The flow was diverted to waste for the first 5 min and last 10 min of the acquisition. The autosampler was maintained at 10 °C and the column was kept at 50 °C. Trypsin-digested β-galactosidase peptides were injected to monitor TOF detector mass calibration after every four samples.

### Data processing

The absolute level of proteins was determined from DIA data handled by Spectronaut (Ver. 13.10.191212.43655, Biognosys, Schlieren, Switzerland) with an internal MDSC spectral library generated by Pulsar (26,184 precursors targeted, 4955 mutated precursors added), default factory DIA settings, and the murine FASTA file from UNIPROT (UP000000589_Mice Reference, release date 8/10/2019, 22,296 protein entries searched) were used. With the output report from Spectronaut, the “Total Protein Approach” was employed for absolute protein level quantitation [62]. In brief, protein quantity was determined from raw intensity values using the formula:

Protein (pmol/mg protein) = (Total intensity/(MW (g/mol) × Total protein intensity)) × 10^9^

Hierarchical clustering was performed using average Euclidean distance method along with principal component analysis (Perseus, Ver. 1.6.14.0) [63]. For comparisons between sample types, protein abundance was transformed to Log 2Fold Change (FC) where FC was determined as lung protein abundance/liver protein abundance (Graphpad Prism, Ver 8.0, La Jolla, CA USA). *P* values were calculated using multiple *t*-tests (where *P* values < 0.05 were deemed significant) and converted to a −Log10 scale to construct a volcano plot with Log2(fold change) data (Graphpad).

Over or under-expressed proteins with a FC > 1.5 or <1.5, respectively (i.e., >0.58 Log_2_FC or <−0.58 Log_2_FC, respectively) that were also statistically significant (*P* value < 0.05) were selected for future pathway analysis and comparisons to RNASeq data. Statistically significant proteins identified by Spectronaut were matched with significant RNASeq data. All statistically significant proteins were also cross-referenced with the drug–gene interaction database (DGIdb) to identify potentially druggable protein targets [64] and further cross-referenced with previously reported RNASeq data [5]. The mass spectrometry proteomics data have been deposited to the ProteomeXchange Consortium via the PRIDE partner repository with the dataset identifier PXD023337 [65].

Samples that were overrepresented in lung but underrepresented in liver were deemed lung targets and vice versa with liver samples. The Target Central Resource Database (TCRD), the web-based data mining platform part of the Illuminate Druggable Genome Project (aka Pharos), was used to further gather information regarding potentially druggable targets and guide future experiments [66]. PHAROs further categorized each protein based on the level of target development into several classes: TDark (virtually unknown or little-known target), TBio (Biological function or Gene Ontology noted), TChem (Biological and Chemical Inhibitors noted), and TClin (approved drugs or ligands with characterized mechanisms of action). Proteins that were consistently expressed across each MDSC type were grouped as being between 0.58 Log2FC and −0.58 Log2FC. Proteins with ratios closer to 1 were designated as MDSC core proteins. Pathway analysis was conducted using STRING (www.STRING-db.org) to identify associations between LM, LuM, or core MDSC proteins with parameters set to identify molecular function using the high confidence threshold (0.7) and hiding disconnected nodes from the overall analysis [67].

### Western blotting

Murine liver (*n* = 5) and lung (*n* = 2) protein extracts (12 μg) were analyzed by reducing SDS-PAGE and transferred to nitrocellulose (Invitrogen). Membranes were washed with Tris-buffered saline (pH 7.6) containing Tween-20 (0.05%) (TBST), blocked for 2 h with 2% (w/v) bovine serum albumin, probed with rabbit anti-Apolipoprotein E (ApoE) antibody (Abcam) and then goat anti-rabbit IgG coupled to horseradish peroxidase (HRP) (Cell Signaling) in TBST with 0.5% (w/v) nonfat dried milk. ApoE was visualized using West Pico PLUS chemiluminescent substrate. Membrane was stripped with Restore stripping buffer (ThermoFisher) and re-probed with anti-glyceraldehyde-3-phosphate dehydrogenase (Gapdh) antibody (Abcam).

## Supplementary information

Supplemental Material-Figure Caption

Supplemental Figure 1-Western Blot

## Data Availability

Proteomic data including raw SWATH files (.wiff), Spectronaut database (.sne), and processed dataset (.xlsx) will be available on ProteomeXchange Consortium via the PRIDE partner repository with the dataset identifier PXD023337.
