# Supplementary material for: Proteomic signatures of myeloid derived suppressor cells from liver and lung metastases reveal functional divergence and potential therapeutic targets
Source: Cell Death Discov. 2021 Sep 4;7:232. doi: 10.1038/s41420-021-00621-x (PMC8418613; doi:10.1038/s41420-021-00621-x)
Supplement: Supplementary file 1 — Supplemental Material-Figure Caption [file 41420_2021_621_MOESM1_ESM.docx]

**Supplementary Information-Figure Legend**

**Figure S1**. Proteomic Validation Blot of Liver Specific Protein ApoE. Murine CD11b+ sorted cells were lysed in RIPA (Liver, n=5;Lung, n=2) buffer before protein determination and western blotting with Gapdh as a loading control.
