# Supplementary figures and images for: Proteomic signatures of myeloid derived suppressor cells from liver and lung metastases reveal functional divergence and potential therapeutic targets
Source: Cell Death Discov. 2021 Sep 4;7:232. doi: 10.1038/s41420-021-00621-x (PMC8418613; doi:10.1038/s41420-021-00621-x)

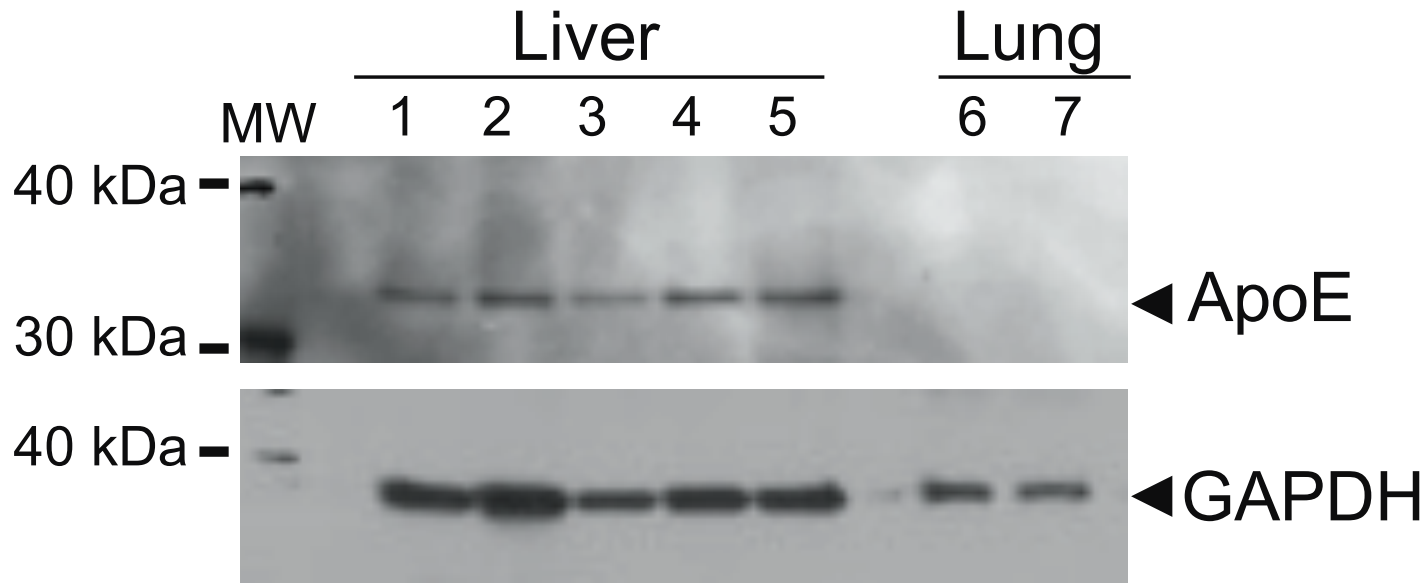

Supplement: Supplementary file 2 — Supplemental Figure 1-Western Blot [file 41420_2021_621_MOESM2_ESM.pdf]
